# Articaine versus lidocaine for third molar surgery: A randomized clinical study

**DOI:** 10.4317/medoral.17148

**Published:** 2011-12-06

**Authors:** Luiz-Carlos Silva, Thiago-de-S Santos, Jadson-A. Santos, Marcelo-C Maia, Carla-G Mendonça

**Affiliations:** 1 Adjunct Professor of Oral and Maxillofacial Surgery and Traumatology, Department of Dentistry, Federal University of Sergipe,Brazil; 2 Master’s student in Oral and Maxillofacial Surgery and Traumatology, Pernambuco Dental School, Brazil; 3 Master’s student in Health Sciences, Center for Postgraduate Program in Medicine, Federal University of Sergipe, Brazil; 4 Graduate student in Dentistry, Federal University of Sergipe, Brazil

## Abstract

Objective: Pain reduction has been the subject of continuous research in the field of oral and maxillofacial surgery since postoperative pain with ranging of intensity and duration may affects the patient submitted in an oral surgical procedure. The aim of present study was to compare the analgesic effectiveness between two different anesthetic solutions (articaine and lidocaine) in third molar surgery. Study Design: A prospective, randomized and clinical study with patients submitted to third molar surgery at two distinct times. The visual analogue scale, the McGill Pain Questionnaire and the analgesic consumption record were used to measure the pain after each surgical time. Results: Duration of surgery, latency, the amount of anesthetic used and analgesic consumption showed clinical differences with highlights of articaine, though statistical significance was not observed (P<0.05). The pain scores indicated similar anesthetic efficacy with both solutions. Conclusion: In the present study no significant differences were observed between lidocaine and articaine in the control of postoperative pain.

** Key words:** Articaine, lidocaine, pain control, lower third molar.

## Introduction

Pain is a protection mechanism of the body to a tissue injury by different stimulations, which transmit a signal to the Central Nervous System (1). Dental pain is usually originated from acute inflammatory nature and it compels the patient for seeking professional help. On the other hand, surgical interventions in dentist office may also induce pain in the postoperative period of previously asymptomatic patients (2).

Pain reduction has been the subject of continuous research in the field of oral and maxillofacial surgery since postoperative pain with ranging of intensity and duration may affects the patient submitted in an oral surgical procedure. Therefore, a method to decrease or eliminate patient pain has its usefulness justified (3). Various medications and analgesic techniques used before the operation can prevent sensitization and postoperative pain. Preemptive analgesia can be obtained with anti-inflammatory, opioid or local anesthetic medications (4). According with Kelly et al. (5), the preemptive analgesia involving local anesthetics has shown to be effective.

The increased availability of local anesthetics has improved interest in research about dental pain control. Nowadays the professional can select from a broad variety of local anesthetic drugs that have the specific properties demanded by the specific case of the patient and the kind of surgical procedure. The concept of local anesthetic action is based on hindering the generation and conduction of nerve impulses. Thus, the impulse is aborted, hindered from reaching the brain and is not interpreted as pain by the patient (4).

Among the local anesthetics, lidocaine is the “standard gold” drug in nowadays. Articaine is outstanding as the local anesthetic indicated for dental procedures and control of postoperative pain (6,7). The proposal of the present study was to compare the use of lidocaine and articaine in the control of postoperative pain for third molar surgery.

## Material and Methods

A prospective,randomized, controlled, parallel group and comparative study design was realized with the aim of observing an association of events. This study was conducted during one year in Aracaju, Sergipe, Brazil. The studied population was selected by spontaneous or referenced demand for services from the Dentistry Department at the Federal University of Sergipe (UFS) in which each patient served as his/her own control. Participation in the study required the patient’s consent in accordance with the recommendations of the National Health Committee and Brazilian Health Department and was approved by the Research Ethics Committee of this institution by number 0025.0.107.000-06. 

A sample of 20 patients ranging from 18 to 30 years old was studied. The inclusion criteria were: patients undergoing removal of bilateral lower jaw third molar surgery in a symmetrical position requiring ostectomy and/or tooth sectioning for extraction. Orthopantomographic radiograms were taken to ensure the similarity of the tooth inclinations and angulations. Third molar had to be class A or B and position 1 or 2, according to Pell & Gregory classification, based on the space relationship of the tooth to the ascending ramus of the mandible and to the occlusal plane of the lower second molar. The Winter’s classification was considered for vertical and/or mesioangular position (8). The exclusion criteria were: systemic disorders or antecedents of complications associated with local anesthetic, patients who were under use of any type of drugs and those that presented any condition that contraindicated the use of sodium dipyrone. 

The surgical and experimental procedures were explained both verbally and in written form and informed consent was obtained before enrollment. Each patient were operated by the same senior oral and maxillofacial surgeon, using the same surgical technique on both sides, with the object of minimizing the discrepancy in the handling of oral issues. 

The choice of the first side to be operated and the group of anesthetic solutions used had been randomly distributed, after a random drawing using the envelope method. Two distinct anesthetic solutions had been used (articaine 4% and lidocaine 2%, both with 1:100.000 of epinephrine – Articaine and Alphacaine, DFL, Brazil), respecting the volume of 4.5ml, with 3.6ml being injected for blocking the inferior alveolar nerve and 0.9ml reserved for blocking the buccal nerve.

To perform the surgical procedure, the material and instruments routinely required for this surgery were used. The surgery to remove the lower third molar followed the standardized technique. Briefly, an “L” shaped incision was made, and a mucoperiosteal flap was elevated. When osteotomy and tooth section were performed on one side, the other side received the same treatment in order to standardize the surgical trauma. All procedures were performed under abundant irrigation with sterilized 0.9% physiological solution. The close of the mucoperiosteal flap was performed with 3-0 silk (Suture needle, TechNew, Brazil). The difficulty of the removal procedure was determined according to the 4 grades by Champbell`s method: (I) simple tooth extraction, (II) bone removal or tooth division, (III) bone removal and tooth division, and (IV) the same as (III) but very difficult (9). For this study, there were considered surgeries that included II and III grades. 

At the end of each surgical procedure, patients received 10 sodium dipyrone pills as supporting analgesic medication for use in case of pain, with instructions to write down the amount and the time when the medication was consumed. 

The duration of the surgical procedure started to count from incision until tooth removal. Surgical procedures exceeding 60 minutes were excluded. When one side exceeds more than 10 minutes the other side, the patient was also excluded. The degrees of difficult extraction, mean duration of surgery, amount of anesthetic used, latency time were also recorded.

After the surgical procedure was performed, the patients were given a chart containing the McGill Pain Questionnaire and Visual Analogue Scale for evaluating postoperative pain, with the explanations of how to proceed with filling it out. The patient proceeded to fill out of McGill Pain Questionnaire on the following morning after surgery and Visual Analogue Scale in the intervals from 2,4,6,12 and 24 hours after surgery according to the instructions.

The McGill Pain Questionnaire is a form that contains some qualitative descriptions of pain (sensory, affective, evaluative and miscellaneous), these being subdivided into 20 groups. From these, the patient only chooses a word from each group, which characterizes the current pain. After data collection, the answers were classified into the above-mentioned standards of pain. 

The Visual Analogue Scale ranges from 0 to 100 (0 is no pain and 100 the worst pain imaginable). The patient was instructed to draw a vertical line to designate a point on the scale to express the degree of pain intensity. According to Collins et al. (10), if a patient records a baseline VAS score in excess of 30 mm they would probably have recorded at least moderate pain on a 4-point categorical scale; in excess of 54 mm and they would probably have recorded severe pain. Using the Visual Analogue Scale and the correlation with a 4-point categorical scale, we determined 4 types of pain intensity: slight, moderate, intense and worst pain. Based on this, we considering less than 30 mm as a slight pain, ranging from 30 mm to 45 mm as moderate pain, ranging from 45 mm to 54 mm as intense pain and over than 54 mm as worst pain. Patient was reevaluated 7 days after surgery and the second surgical time was performed.

All statistical analysis was done with the Bioestat statistical analysis system. Data were presented as the mean with their standard deviations and 95% confidence intervals for the mean where applicable. Demographic data, duration of operation and amount of local anesthetic used were evaluated with paired student’s t test. The difference in pain scores were analyzed with student’s t test. The analgesic consumption were analyzed by X2 with Yates correction and the McGill Questionnaire was analyzed by descriptive statistical. Differences from baseline preoperative values in pain measurements were normally distributed (Komogorov-Smirnov Test). 

## Results

Using the principal variable, the visual analog scale (VAS) scores for postoperative pain, and considering a difference of 20 mm as clinically significant (estimated mean standard deviation 15 to 25 mm) the sample size was calculated as 20 patients in each group, that means in this study 20 patients, each side of patient with a different local anesthetic, considering a type II error of 0.20 and a type I error of 0.05, and a statistical power of 80%.

Of the 24 patients entered into the study two were excluded from the analysis due to incomplete pain diary form, and the others 2 patients did not return to the second surgery. Due to the within-subject spit mouth study design with the patient constituted his/her own control, the influence of sex and weight, as well as others demographic factors, had little effect on treatment outcome.

Among the studied sample of 20 patients, ranging from 18 to 30 years old, we found a mean age of 23.25 years.

**Table 1:**
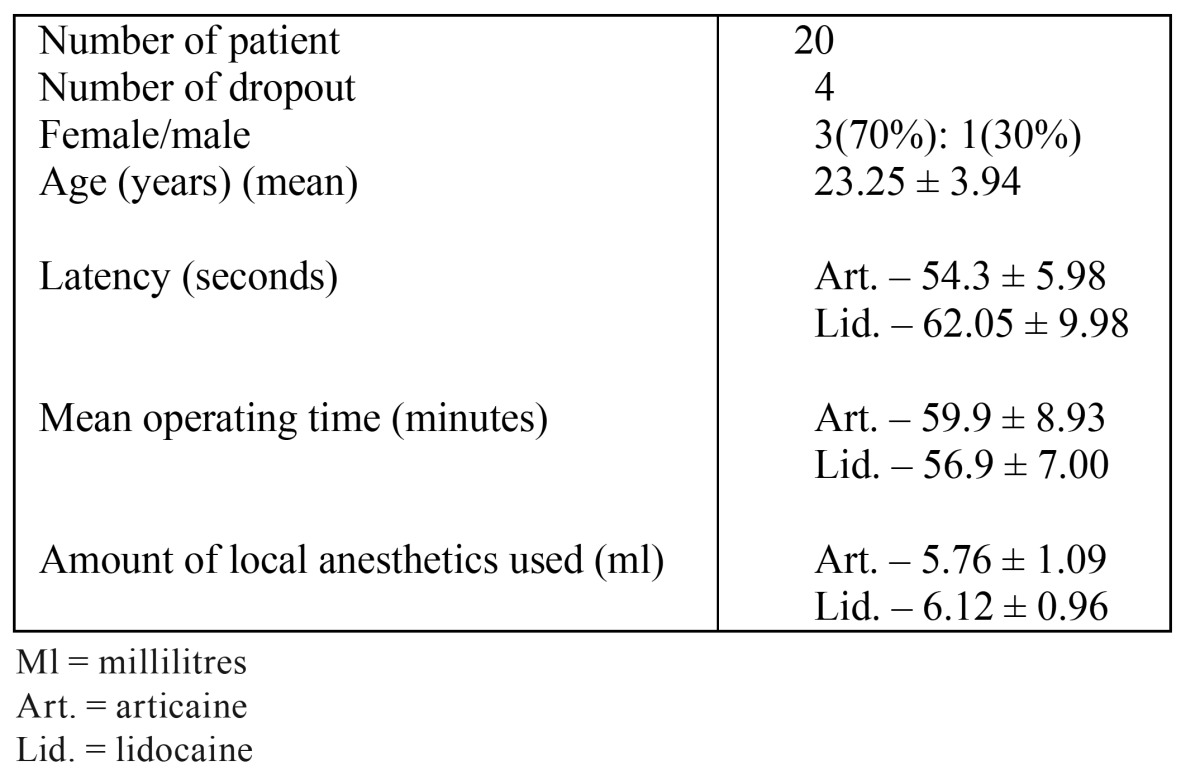
Baseline characteristics.

**Figure 1:**
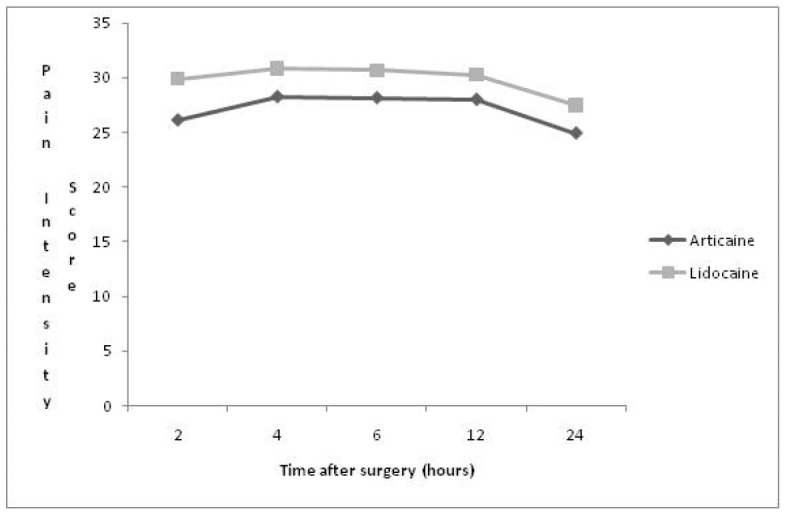
Time after surgery hours.

**Table 2:**
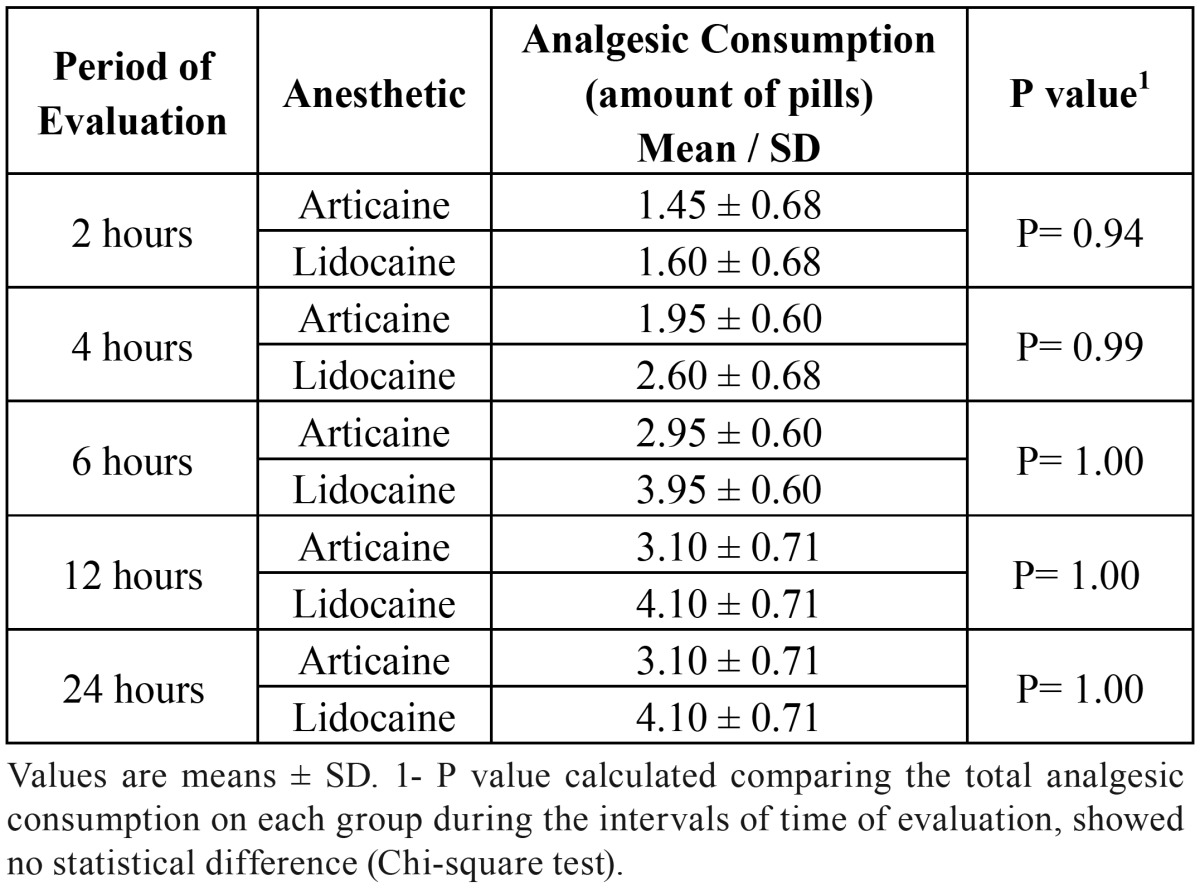
Amount of analgesic used.

A prevalence of women was recorded, in a ratio of 3:1, no significant between-group differences were found in the mean duration of surgery, amount of local anesthetic used ( Table 1), and the degree of difficult of extraction, characterizing the baseline of this study. The results showed a prevalence of pain of slight and moderate intensity.

In the period of 2 hours evaluation, all patients referred pain, irrespective of the type of anesthetic used. The number of patients reported referring pain using lidocaine was the double (30%) between the levels of intense and worst, when compared with the patients who had used articaine (15%) (mean score- 26.1 mm / 29.9 mm for lidocaine), but the difference was not significant (p = 0.46, paired T-test). The mean pain intensity scores throughout 24-h investigation period are shown in (Fig. 1).

No significant differences were found in the period of 4 hours for both anesthetic solutions, at all levels of pain quantification (Art – 28.25 mm; Lid – 30.85 mm – mean score/ p = 0.41). The same occurred with the 6-hour period (Art – 28.15 mm; Lid – 30.7 mm - mean score/ p = 0.33); it should be pointed out that the worst level of pain was measured by 1 patient who used lidocaine, which did not occur when the articaine solution was used. Similar data were recorded in the 12 post-operative hours (Art – 28.00 mm; Lid – 30.25 mm – mean score/ p= 0.37).

**Table 3:**
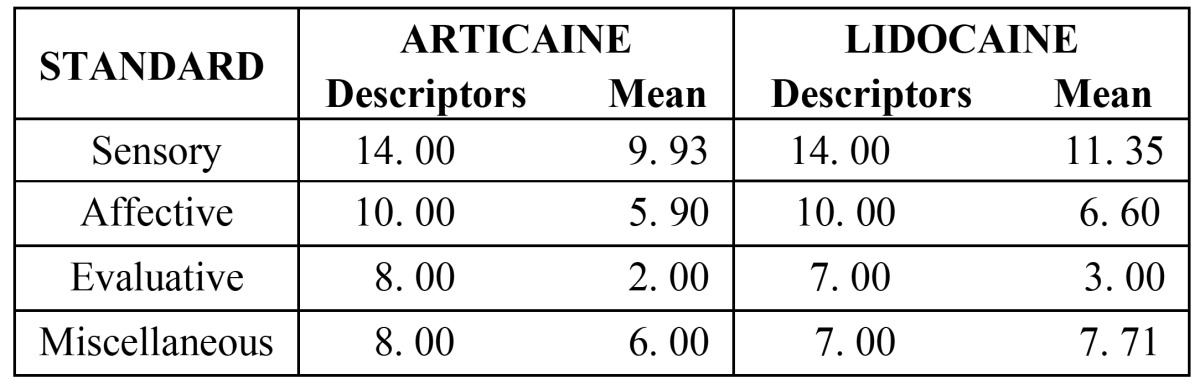
The McGill Questionaire of Pain.

One day after surgery, it was observed that a lower number of patients who had used articaine referred pain, and also presented slight pain intensity (4 patients). When analyzing the data obtained by the Visual Analogue Scale, the presence of pain and evaluated intensity could be determined for the type of anesthetic used, and no significant difference between the results presented for these anesthetic solutions was found.

At all the evaluation times there was no quantitative difference in the number of pills who had used after surgery, considering both anesthetic solutions ( Table 2). 

The Mc Gill Pain Questionnaire generated the following measures: number of chosen descriptors and pain index. The number of chosen descriptors corresponds to the words that the sick person chooses to explain pain. The standards classified as sensory and affective were the pain evaluation descriptors which were most frequently mentioned by the patients in this study ( Table 3).

## Discussion

The age of the sample consisted of patients ranging from 18 to 30 years of age, which can be verified in the majority of comparative studies related anesthetic and third molar surgery (9,11,12).

A gradual reduction in pain intensity was identified in the evaluated times, as well as in the percentage of patients with postoperative pain. This reduction is justified by the peaks of postoperative pain that generally occur in first 8, 12 hours (12). Therefore, nowadays local anesthetic solutions with long-duration are more frequently used, in an effort to reduce the consumption of analgesics in the post-surgical period (6,13,14).

In the present study, no difference in postoperative pain was found between the two tested anesthetic solutions. This contradicts the conclusions found in the study of Ruprecht and Knoll-Kohler (15) where they evaluated the same anesthetics solutions of the present study and it was observed articaine presented a more extended, faster anesthetic than lidocaine. In the work of Sierra-Rebolledo et al. (16) it was also observed that postoperative pain control with articaine was more satisfactory.

Our findings were similar to Malamed et al. (17), whose there was no significant differences between articaine and lidocaine in pain intensity measured by the Visual Analogue Scale. Vahatalo et al. (18) evaluated the periods of latency and duration of 4% articaine and 2% lidocaine (with 1:100.000 epinephrine) observed that articaine presented a longer duration, about 45 seconds more than lidocaine, pointing out that this difference is not statistically significant.

In a general evaluation, the similarity of effectiveness of the anesthetics used in postoperative pain control is well established, being based on the patient’s analgesic consumption that presented an equivalent ratio. When considering that pain manifests more intensely in first 12 hours with the peak generally being observed around the 6th, 8th hours (19-22), this affirmation was found to be valid in this study, since there were periods of evaluation when a higher number of pills was used by the patients.

No significant differences were observed between the tested anesthetic solutions for postoperative pain control in third molar surgery, with the use of the Visual Analogue Scale, the McGill Pain Questionnaire and the consumption of analgesics as published in Evans et al. (23) report about an evaluation of the anesthetic efficacy of articaine 4% and lidocaine 2%, both with 1:100.000 of epinephrine in maxillary lateral incisors and first molars. 

Research based on these pain control parameters is difficult to standardize, due to the pain threshold of each patient, as well as degree of difficulty of patients to understand the instructions for filling out the questionnaire. It is suggested that further research must be conducted with higher number of patients.

## References

[1] Hunter JP, Simmonds MJ (2010). Pain: putting the whole person at the centre. Physiother Can.

[2] Fernandes MJ, Ogden GR, Pitts NB, Ogston SA, Ruta DA (2009). Incidence of symptoms in previously symptom-free impacted lower third molars assessed in general dental practice. Br Dent J.

[3] Neal JA, Welch TB, Halliday RW (1993). Analysis of the analgesic efficacy and cost-effective use of long-acting local anesthetics in outpatient third molar surgery. Oral Surg Oral Med Oral Pathol Oral Radiol Oral End.

[4] Pozos-Guillen A, Martinez-Rider R, Aguirre-Banuelos P, Perez-Urizar J (2007). Pre-emptive analgesic effect of tramadol after mandibular third molar extraction: a pilot study. J Oral Maxillofac Surg.

[5] Kelly DJ, Ahmad M, Brull SJ (2001). Preemptive analgesia II: recent advances and current trends. Can J Anaesth.

[6] Dionne RA, Wirdzek PR, Fox PC, Dubner R (1984). Suppression of postoperative pain by the combination of a nonsteroidal anti-inflammatory drug, flurbiprofen, and a long-acting local anesthetic, etidocaine. J Am Dent Assoc.

[7] Lima-Júnior JL, Dias-Ribeiro E, de Araújo TN, Ferreira-Rocha J, Honfi-Júnior ES, Sarmento CFM (2009). Evaluation of the buccal vestibule-palatal diffusion of 4% articaine hydrochloride in impacted maxillary third molar extractions. Med Oral Patol Oral Cir Bucal.

[8] Yuasa H, Kawai T, Sugiura M (2002). Classification of surgical difficulty in extracting impacted third molars. Br J Oral Maxillofac Surg.

[9] Campbell WI, Kendrick RW, Fee JP (1998). Balanced pre-emptive analgesia: does it work? A double-blind, controlled study in bilaterally symmetrical oral surgery. Br J Anaesth.

[10] Collins SL, Moore RA, McQuay HJ (1997). The visual analogue pain intensity scale: what is moderate pain in millimetres?. Pain.

[11] Katyal V (2010). The efficacy and safety of articaine versus lignocaine in dental treatments: a meta-analysis. J Dent.

[12] Nayyar MS, Yates C (2006). Bupivacaine as pre-emptive analgesia in third molar surgery: Randomised controlled trial. Br J Oral Maxillofac Surg.

[13] Hyrkäs T, Ylipaavalniemi P, Oikarinen VJ, Paakkari I (1994). Effective postoperative pain prevention through administration of bupivacaine and diclofenac. Anesth Prog.

[14] Rosenquist JB, Rosenquist KI, Lee PK (1988). Comparison between lidocaine and bupivacaine as local anesthetics with diflunisal for postoperative pain control after lower third molar surgery. Anesth Prog.

[15] Ruprecht S, Knoll-Köhler E (1991). A comparative study of equimolar solutions of lidocaine and articaine for anesthesia A randomized, double-blind, cross-over study. Schweiz Monatsschr Zahnmed.

[16] Sierra Rebolledo A, Delgado Molina E, Berini Aytís L, Gay Escoda C (2007). Comparative study of the anesthetic efficacy of 4% articaine versus 2% lidocaine in inferior alveolar nerve block during surgical extraction of impacted lower third molars. Med Oral Patol Oral Cir Bucal.

[17] Malamed SF, Gagnon S, Leblanc D (2000). Efficacy of articaine: a new amide local anesthetic. J Am Dent Assoc.

[18] Vähätalo K, Antila H, Lehtinen R (1993). Articaine and lidocaine for maxillary infiltration anesthesia. Anesth Prog.

[19] Chapnick P, Baker G, Munroe C (1980). Bupivacaine anaesthesia in oral surgery. J Can Dent Assoc.

[20] Seymour RA, Rawlins MD, Rowell FJ (1982). Dihydrocodeine induced hyperalgesia in pos-operative dental pain. Lancet.

[21] Cooper SA, Beaver WT (1976). A model to evaluate mild analgesics in oral surgery outpatients. Clin Pharmacol Ther.

[22] Campbell WI, Kendrick RW (1997). Pre-emptive analgesia using local anaesthesia: a study in bilaterally symmetrical surgery. Br J Anaesth.

[23] Evans G, Nusstein J, Drum M, Reader A, Beck M (2008). A prospective, randomized, double-blind comparison of articaine and lidocaine for maxillary infiltrations. J Endod.

